# Emotion in Stories: Facial EMG Evidence for Both Mental Simulation and Moral Evaluation

**DOI:** 10.3389/fpsyg.2018.00613

**Published:** 2018-04-30

**Authors:** Björn 't Hart, Marijn E. Struiksma, Anton van Boxtel, Jos J. A. van Berkum

**Affiliations:** ^1^Languages Literature and Communication, Utrecht Institute of Linguistics OTS, Utrecht University, Utrecht, Netherlands; ^2^Cognitive Neuropsychology, Social and Behavioral Sciences, Tilburg University, Tilburg, Netherlands

**Keywords:** emotion, language processing, language comprehension, embodiment and grounded cognition, evaluation, narrative comprehension, facial EMG

## Abstract

Facial electromyography research shows that corrugator supercilii (“frowning muscle”) activity tracks the emotional valence of linguistic stimuli. Grounded or embodied accounts of language processing take such activity to reflect the simulation or “reenactment” of emotion, as part of the retrieval of word meaning (e.g., of “furious”) and/or of building a situation model (e.g., for “Mark is furious”). However, the same muscle also expresses our primary emotional evaluation of things we encounter. Language-driven affective simulation can easily be at odds with the reader's affective evaluation of what language describes (e.g., when we like Mark being furious). To examine what happens in such cases, we independently manipulated simulation valence and moral evaluative valence in short narratives. Participants first read about characters behaving in a morally laudable or objectionable fashion: this immediately led to corrugator activity reflecting positive or negative affect. Next, and critically, a positive or negative event befell these same characters. Here, the corrugator response did not track the valence of the event, but reflected both simulation and moral evaluation. This highlights the importance of unpacking coarse notions of affective meaning in language processing research into components that reflect simulation and evaluation. Our results also call for a re-evaluation of the interpretation of corrugator EMG, as well as other affect-related facial muscles and other peripheral physiological measures, as unequivocal indicators of *simulation*. Research should explore how such measures behave in richer and more ecologically valid language processing, such as narrative; refining our understanding of simulation within a framework of grounded language comprehension.

## Introduction

Imagine this: you walk up to your car and see a kid scratch it with a key and run off. There is a good chance that you would be furious and frown at least a little bit as part of the expression of that anger. Facial expression is part of our emotional evaluation of the world around us (Darwin et al., 1998; Keltner and Ekman, 2000). Because we reliably frown more when we evaluate things as being negative and less when we deem something positive, the *corrugator supercilii* muscle is especially useful as an indicator of our emotional evaluation of things, and of how we express that evaluation to others. Using surface facial electromyography (EMG), we can accurately record corrugator activity. Its strong negative linear relationship to emotional valence, ranging from positive to negative, makes it a reliable indicator of the emotional significance of a given stimulus (Tassinary et al., 2000; Larsen et al., 2003).

Now consider the following sentence: “Mark is furious when he walks up to his car and sees a kid scratch it with a key and run off.” A number of studies have shown that simply processing affectively salient language, even if it describes some fictional character's anger at their fictional car being keyed, will *also* evoke corrugator activity (e.g., Foroni and Semin, 2009; Glenberg et al., 2009; Niedenthal et al., 2009). These studies interpret the activity of this facial muscle within the framework of grounded cognition as language-driven *simulation* of what is being referred to.

Simulation, in these cases, is taken to denote the neural reactivation of experiential traces stored from earlier perceptual, affective, and motor experience with the world (e.g., Barsalou, 2008). Evidence for language-driven simulation has come from various types of studies. For perceptual language, for example, behavioral studies showed a facilitation effect for verifying whether a picture of an eagle with wings outstretched in fact contained an eagle. That is to say, participants were faster after a sentence describing it in the air (e.g., “the ranger saw the eagle in the sky”) compared to after a sentence describing it sitting (e.g., “the ranger saw the eagle on the post”) (Zwaan et al., 2002; Zwaan and Pecher, 2012). These results indicated perceptual simulation of the eagle in a particular position was part of language comprehension. Neuroimaging evidence provided further support for such perceptual simulation. For instance, Simmons et al. (2007) found that a verbal object-property verification task related to color activated the same areas of the brain involved in color perception, while a verification task related to movement did not. While there is an ongoing debate regarding the precise nature, and necessity, of simulation in language comprehension (e.g., Barsalou, 2016; Leshinskaya and Caramazza, 2016), converging evidence has led to a relative consensus on its existence. This consensus goes beyond perceptual language alone and includes affective language (Vigliocco et al., 2009). Much of the evidence for affective simulation comes from facial EMG studies, such as cited above, showing EMG activity congruent with the basic affective valence of linguistic stimuli. One notable study by Havas et al. (2010) even demonstrated that paralysis of the corrugator with Botox led to slower processing of negatively valenced sentence. Within the grounded cognition framework, corrugator activity is most sensibly interpreted as a downstream consequence of the *neural* simulation of affect (rather than as reflecting a critical role for muscle activity *itself* in language processing).

**Figure 1:**
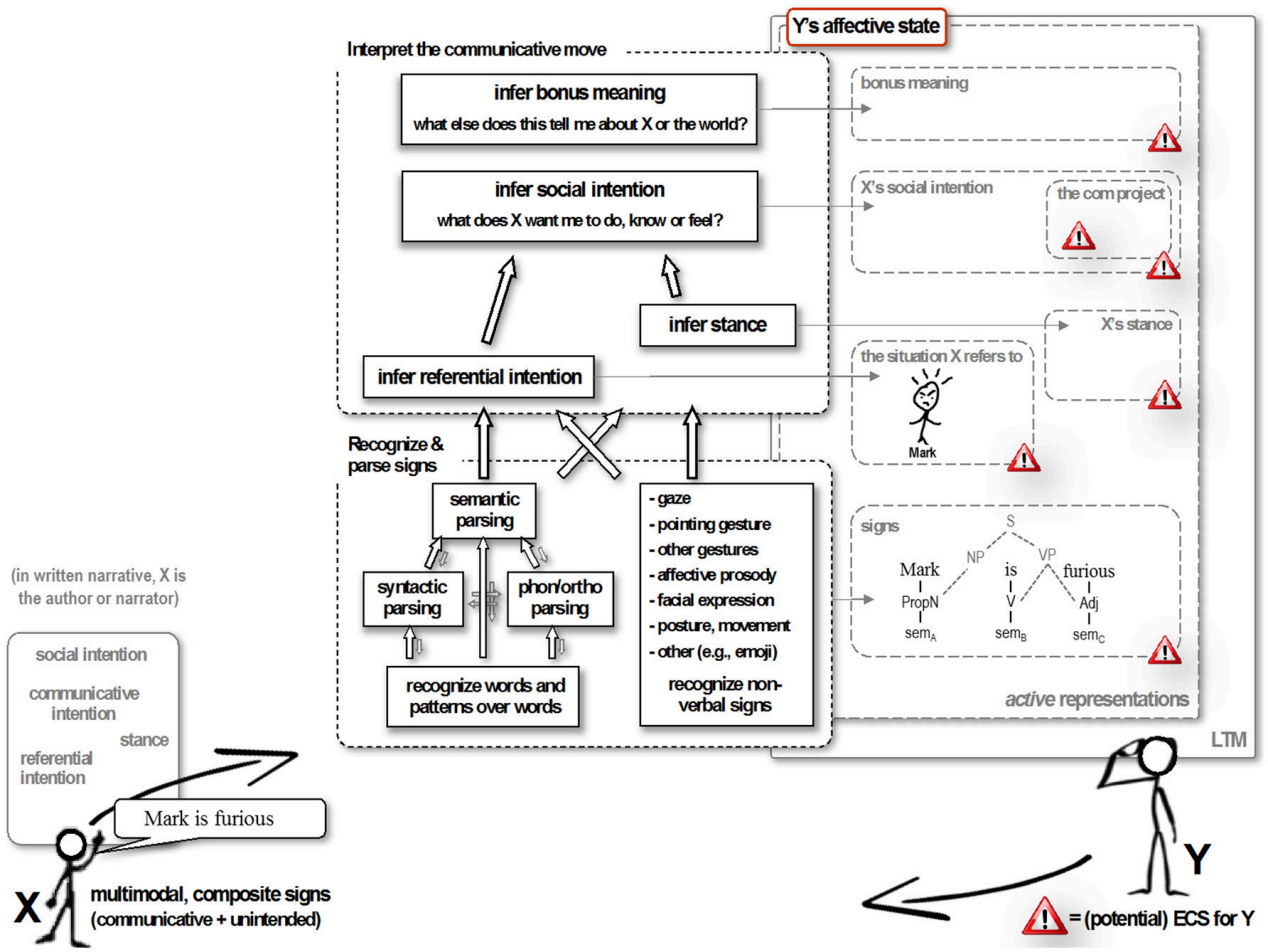
Example processing of “Mark is furious” in the Affective Language Comprehension model. Mental processes and the associated retrieved or computed representations are expanded for addressee Y only. Y's computational processes draw upon (and add to) long-term memory traces, and involve currently active dynamic representations that reflect what is currently retrieved from LTM, composed from elements thereof and/or inferred from context, in response to the current communicative move. Y's active representations can be conscious or unconscious. For narratives presented on screen in a laboratory experiment without a foregrounded author or narrator, stance and social intention are presumed to be irrelevant. ECS, emotionally competent stimulus; com project, communicative project. See Van Berkum (2018a,b) for detailed explanation.

A recent broad model of the interfaces between language comprehension and emotion, the *Affective Language Comprehension (ALC)* model (Van Berkum, 2018a,b), can help us discuss the various ways in which a sentence like “Mark is furious when…” can generate an emotional state, as well as the associated facial expression, in the reader. As shown in Figure 1, language comprehension is assumed to involve a decoding process in which comprehenders retrieve and grammatically combine word meanings (and recognize other signs as well, such as, in writing, a word printed in italics, or an exclamation mark), and an interpretive process in which comprehenders infer the speaker's various intentions in the context at hand. Following Tomasello (2008), the latter includes working out what situation the speaker is referring to, and understanding what the speaker wants to achieve by this. Importantly, in the ALC model, the various representations retrieved or constructed as part of these processes can in principle *all* serve as emotionally competent stimuli (ECSs), i.e., elicit a conscious or subconscious affective response.

As can be seen in Figure 1, the model suggests that language-driven affective *simulation* might feature as part of two very different subprocesses contributing to language comprehension. One is the recognition and parsing of the composite sign “Mark is furious,” which involves retrieving the meanings of each of the words from long-term memory (sem_A_, sem_B_, and sem_C_ in Figure 1) and combining them in line with grammatical constraints. To the extent that the meaning of a word like “furious” includes traces of actually being furious (e.g., Foroni and Semin, 2009; cf. Pülvermüller, 2013), retrieving that lexical-conceptual meaning can be said to involve affective simulation. A second and theoretically distinct subprocess that might feature language-driven affective simulation is inferring the referential intention of the author (or narrator), which in the case at hand involves identifying the particular story character designated by “Mark” in the evolving situation model for the narrative, and updating the situation model in ways suggested by the semantics of the predicate “is furious”. To the extent that this situation model updating comes down to actually imagining a furious specific character, affective simulation could be part of the construction process (cf. Zwaan and Radvansky, 1998; Zwaan and Kaschak, 2008; Zwaan, 2014).

Language-driven affective simulation can in in principle thus feature in two different subprocesses that are needed to arrive at a representation of what is being described in a narrative: the representation of sign meaning, and the construction of a situation model. According to the ALC model, affective *evaluation* is something quite different, and involves how the comprehender emotionally *reacts to* the various representations that become available. This can be an evaluative response to what is in the situation model, to the author's (or narrator's) inferred stance and social intention, and to additional inferences triggered by any of this. To return to the example, affective evaluation reflects how the comprehender *feels about* such things as Mark being furious (in the real world or a fictional one), and, in richer real-world contexts, about why somebody is informing him or her about this fact in a particular way. Such reactive evaluation of what happens around us (or is talked about in, e.g., gossip or stories) is why we have emotions in the first place.

If the neural systems involved in natural emotional evaluation (e.g., *your* outrage over some situation or event) are also used as part of such language-driven *simulation* (e.g., reading about *somebody else's* outrage over some situation or event), this raises an interesting question: what happens when the same systems are recruited to conflicting ends? The issue does not easily come up in grounded language processing experiments that use only single words or short sentences (e.g., “furious,” or “Mark is furious”), because, although such simple materials afford simulation, they do not necessarily elicit a lot of evaluation. Moreover, the evaluation that is elicited by such limited stimuli is likely to always be congruent with language-driven simulation. However, if we scale up complexity by placing such materials in a richer discourse context (Van Berkum, 2008; Zwaan, 2014), evaluation may covary or conflict with simulation. Consider again the example of reading about Mark's car getting keyed; as we process the language describing Mark's anger and construct the associated situation model, neural simulation in support of language comprehension will result in corrugator activity that reflects negative affect. However, if we feel that Mark is a bad person, we may well evaluate his negative affect as something he deserved, so as positive. While it is well-established that Schadenfreude does in fact occur in such cases (e.g., Feather and Nairn, 2005; Singer et al., 2006; Leach and Spears, 2009) and that this can also influence facial muscle activity recorded using EMG (Cikara and Fiske, 2012), to our knowledge no research has been done on how such affective evaluation meshes with language-driven simulation. Our experiment focuses specifically on how the conflicting demands that affective evaluation and language-driven affective simulation make on emotion-relevant motor systems play out, and how that is reflected in the corrugator activity.

In our experiment we explored the potential conflict between language-driven simulation and emotional evaluation by measuring corrugator activity over two critical segments in short narratives. First, we manipulated the moral status of a main character by having this protagonist act either morally or immorally. Second, we manipulated a critical event where the protagonist experiences something that is positive or negative *to them*. The character morality manipulation was designed to render the character moral (“good”) or immoral (“bad”) to the average reader, and to as such create the basis for a differential, character-dependent moral evaluation of subsequent good or bad critical events befalling the character. We assumed that good events happening to moral characters would primarily be evaluated as fair and lead to corresponding positive emotions (e.g., a sense of justice), whereas bad events happening to those characters would primarily be evaluated as unfair and lead to corresponding negative emotions (e.g., moral indignation, anger, pity). Critically, we assumed that how readers evaluate those same events would change dramatically when these events would befall *immoral* characters, with good events happening to those characters primarily evaluated as unfair, leading to related negative emotions (e.g., moral indignation, anger, irritation), and bad events happening to those characters primarily evaluated as fair, leading to positive emotions (e.g., *Schadenfreude*, a “serves-you-right” sense of justice). In Table 1 above, an example narrative illustrates the design of our study, as well as the specific timing of presenting the various fragments.

**Table 1:** Example narrative, also illustrating trial structure and timing.

Baseline	Neutral distractor image			3 s
Introduction	Mark is driving through the pouring rain, on his way to his mother. He's still in the inner city and big puddles have formed. It's been raining non-stop since yesterday. Some streets are practically flooded. There are few cars on the road and fewer bicycles and pedestrians still. Mark is headed for a giant puddle and spots a pedestrian on the sidewalk.			18 s
Character Morality (moral/immoral)	Mark slows down to avoid the puddle, making sure he doesn't splash the pedestrian.	OR	Mark accelerates through the puddle on purpose to create a big splash and soak the pedestrian.	5 s
Continuation	Once outside the city he is driving along on the freeway. There still isn't a lot of traffic and Mark is enjoying the landscape and the drive. He's got the radio on full blast and sings along loudly. When he glances at the dashboard to adjust the channel he spots a warning light. He forgot to put petrol in the car and has been running on empty for a while.			15 s
Critical Event (positive/negative for the character)	Mark is happy when he immediately spots a petrol station and he avoids being stranded.	OR	Mark is frustrated when there isn't a petrol station in sight and he becomes stranded by the roadside.	5 s
	Press “space” to continue to the next story			

On the assumption that participants are in a neutral or otherwise moderate affective state as they read the introduction, our predictions for corrugator activity at the subsequent character morality segment are straightforward: increased corrugator activity (frowning) for immoral actions, but a decrease for moral actions (relaxation). Although also of interest in itself, such a differential response would above all suggest that the character morality manipulation was successful, and that the stage would be set for subsequent events.

The critical predictions concern what happens when readers subsequently read about good or bad events befalling the character. With moral characters, negative events are expected to clearly increase corrugator activity, because of a negatively valenced simulation of the character's state, a negatively valenced evaluation by the reader, or a combination of the two. For similar reasons, positive events befalling moral characters are expected to relax the corrugator, because of a positively valenced simulation, a positively valenced evaluation, or both. With *immoral* characters, however, simulation and evaluation valence will conflict, at least for the average reader: something *bad* happening to an immoral character is negative for the character but positive for the reader, and something *good* happening to an immoral character is positive for the character but negative for the reader. Hence, with immoral characters, language-driven simulation should in principle recruit the neural systems that control the corrugator to simulate one valence (positive or negative) while evaluation should in principle recruit those same systems to express the opposite valence. The outcome of this conflict is the main focus of the experiment.

**Figure 2:**
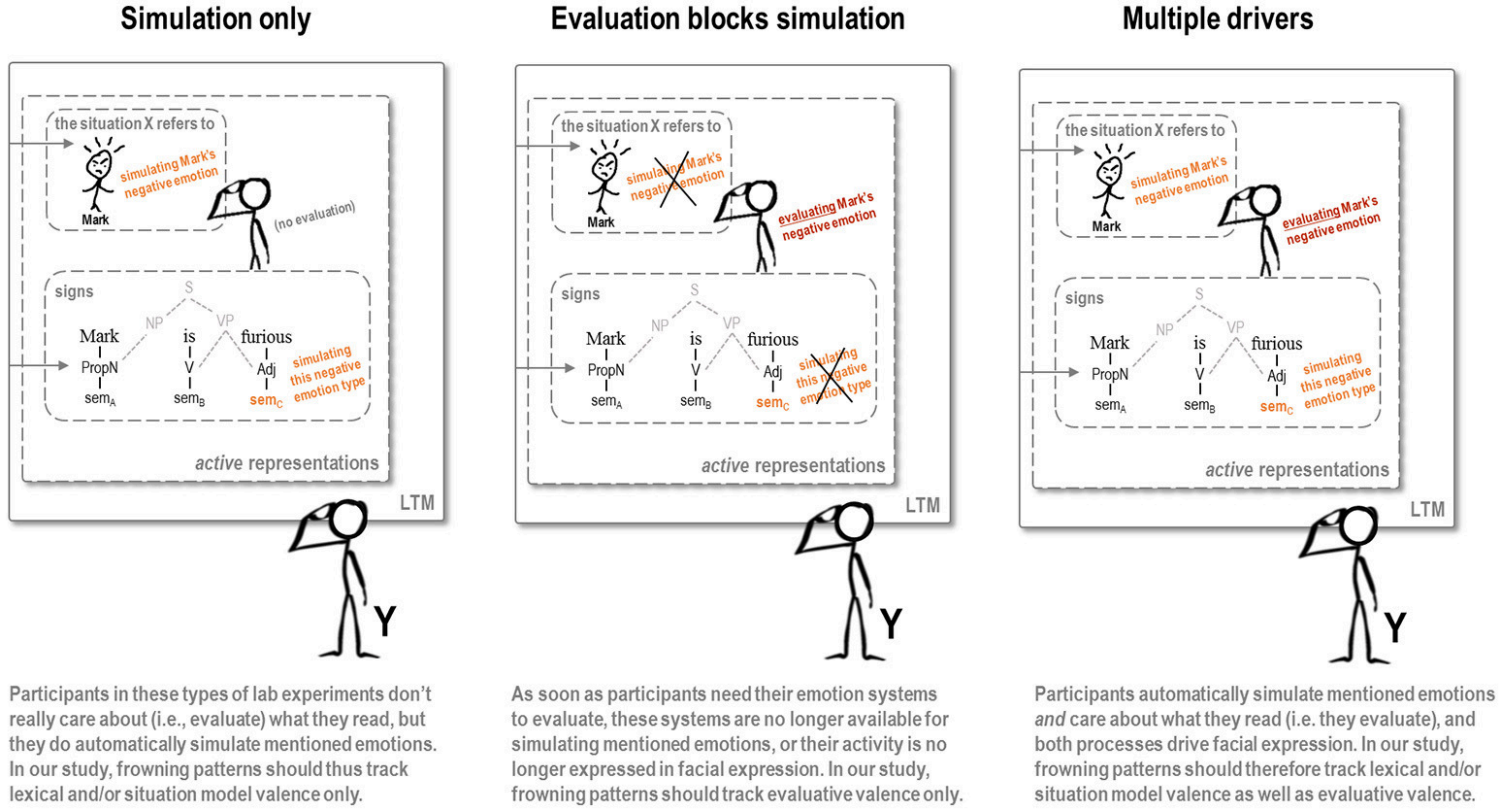
Three possible arrangements of how language-driven affective simulation and evaluation drive the corrugator muscle.

Based on the ALC model, we considered three possible accounts to predict corrugator activity at the critical event, schematically depicted in Figure 2. The first is one where, during narrative fiction reading, language-driven simulation totally captures the corrugator, and does not reflect evaluation. This *simulation-only* account predicts that even in the face of oppositely valenced evaluation (i.e., good or bad things befalling an immoral protagonist), corrugator activity would simply show the simulation involved in constructing a situation model (imagining the protagonist as frustrated or happy) and/or the simulation of lexical meaning (“frustrated,” “happy”) in the service of such construction. We deem this account rather unlikely, in part because of the evolutionary significance of narrative in moral and other social-affective evaluations (notably in gossip, Dunbar, 2004), and because of the roles that affective evaluation and the accompanying overt facial expressions play in socially responding to interpersonal narrative (with facial expression usually seen as a *constituent* of such emotion, see Van Berkum, 2018a,b). Furthermore, as simulation is itself grounded in such primary affective evaluation, it would be peculiar to predict that in language comprehension, simulation prevails over “the real thing.” We include the *simulation-only* account as a logical possibility, though, in part because it can be taken to represent the tacit assumption made in much grounded language processing research.

The second account under consideration holds that, in line with the role of narrative (e.g., gossip) in moral evaluation, the emotional response to the perceived fairness of the event completely captures the corrugator such that the neural systems controlling it are no longer available for language-driven simulation of lexical meaning or a character's emotion. This *evaluation-blocks-simulation* account predicts that the moral status of the protagonist determines the ultimate evaluative valence of a critical event in terms of *fairness*, causing the corrugator responses to critical events to “flip” when those events befall immoral rather than moral characters. For instance, relative to positive events, negative events should lead to increased corrugator activity (i.e., negative affect) when they happen to a morally good character, but to *de*creased corrugator activity (i.e., positive affect) when they happen to a bad character. This account makes the reasonable assumption that, although the neural systems that control facial muscles might be free for simulating emotion as long as people don't care about what they read, those systems will immediately be recruited in the service of *real* evaluative emotion as soon as a narrative describes something worthy of evaluation.

The third account we consider is one in which evaluation and simulation both determine corrugator activity. This *multiple-drivers* account predicts that simulation and evaluation both leave traces in the activity of the corrugator as indexed by EMG. Because the drivers may interact in ways that are difficult to lay out in advance, the exact pattern of results is hard to predict. The one clear prediction, though, is that the corrugator EMG patterns cannot be explained in terms of one of the simpler models laid out before.

The strength of the effects hypothesized under these three accounts could arguably vary as a result of individual differences. We included two questionnaires that measure the tendency of people to experience emotions in response to narratives and their tendency to empathize and/or sympathize with the emotions of others. For the former we use the Transportability questionnaire (Dal Cin et al., 2004), measuring the degree to which people are readily transported into narratives. Research shows that transportation influences, among other things, the intensity of our emotional experience of a narrative (e.g., Green et al., 2004). The second questionnaire, the Adolescent Measure of Empathy and Sympathy (AMES; Vossen et al., 2015), measures three components of pro-social emotion: affective empathy (“feeling what another feels”), cognitive empathy (“understanding what another feels”), and sympathy (“feeling for the other”).

## Methods

### Participants

Sixty students (47 female, age range 18–30, *M* = 21.08, *SD* = 3.43) recruited from the participant pool of the UiL OTS participated in exchange for financial compensation (€ 12,-). All participants were native speakers of Dutch, without a diagnosis of dyslexia, without Botox injections in the face and with normal or corrected-to-normal vision. At the time this research was conducted, the research institute where it took place did not yet have an Ethics Committee (Institutional Review Board), and institute guidelines did not require any other formal ethics approval. Because there is no medical aim involved, the research at hand also did not fall under the scope of national legislation requiring medical ethics review (The Dutch *WMO, Medical Research Involving Human Subjects Act*). Research procedures complied with *The Netherlands Code of Conduct for Academic Practice*, as well as with the *Declaration of Helsinki*. In line with the latter, all of our participants gave written informed consent, based on an elaborate informed consent form detailing the nature of the materials and the procedure, and emphasizing their right to withdraw consent at any time during the experiment without being required to provide a reason, and without losing their right to financial compensation. The informed consent form (in Dutch) is available upon request from the corresponding author.

### Design

Our experiment had a 2 × 2 design: Character Morality (moral vs. immoral) and Critical Event (positive vs. negative). The design was fully crossed and implemented within subjects. Our main dependent variable was *corrugator supercilii* activity. We included measures of individual differences concerning transportability and empathy and sympathy as possible factors that would influence the effect our manipulations had on corrugator activity. The stimulus design is illustrated in Table 1 above and discussed further below.

### Materials

#### Narrative stimuli

Sixty-four short narratives were created for the experiment according to the structure outlined in Table 1 above, each in four variants based on our 2 × 2 (morality x event) design. The character morality manipulation was pre-tested in a different group of 38 students (35 female), recruited from the same participant pool and similar to the experiment group. The pre-test participants were divided into two groups and each read half (32) of the stories up to and including the moral manipulation. They were first asked to rate, on a 7-point scale, how prosocial (1) or antisocial (7) the actions of the protagonist were, and, second, how expected (1) or unexpected (7) the actions of the protagonist were. Moral actions were considered more prosocial (*M* = 1.69, *SD* = 1.04) than immoral actions (*M* = 5.99, *SD* = 1.04). Moral actions were also considered slightly more expected (*M* = 3.17, *SD* = 1.57) than immoral ones (*M* = 5.16, *SD* = 1.56). In the experiment, each narrative was preceded by a baseline measure which included the presentation of a neutral distractor image of a path in a forest. The reason for including an image rather than, for instance, a fixation cross was to reduce the degree to which participants' minds would wander and thus skew the baseline facial EMG recording.

We created four pseudorandomized stimulus lists with 64 narratives each, such that (a) every narrative occurred once in one of four variants in each list, (b) participants would see 16 narratives in each of the four conditions, 8 with a male and 8 with a female protagonist, (c) average item properties in each list were similar in terms of pro-sociality (and expectedness as its inevitable correlate, (d) two lists had the reverse order of two other lists, and (e) each narrative occurred with both male and female protagonists across the four different lists, with the exception of 9 narratives that had fixed gender due to stereotypical behavioral expectations. Dutch stimulus materials are available upon request.

#### Questionnaires individual differences

We included a Transportability questionnaire (Dal Cin et al., 2004), measuring the degree to which people are readily transported into narratives through agreement (on a scale of 1–9) with statements such as such as “I find I can easily lose myself in the story” and “I am often emotionally affected by what I've read.” The second questionnaire, the Adolescent Measure of Empathy and Sympathy (AMES; Vossen et al., 2015)^1^, measures three components of pro-social emotion: affective empathy (e.g., “when a friend is angry about something, I become angry too”), cognitive empathy (e.g., “I can tell when a friend is angry, even when he/she tries to hide it”), and sympathy (e.g., “I feel bad for a friend when he/she is sad”).

### Procedure and data acquisition

After reading and signing an informed consent form, participants received verbal instructions. With regards to the reading of the stimuli, they were instructed to read them silently as they would when reading a book. They were not given specific instruction about what to look for or pay attention to. In a separate room, stimuli were presented on a screen at a distance of approximately 60 cm. Stimuli were presented in automatically timed segments as outlined in Table 1, in white Times New Roman (24 pt.) on a black background. Participants read 64 narratives in total, preceded by 2 practice trials. Presentation rate of trials was self-paced by pressing space bar between stories with two fixed longer pauses during the experiment. Facial EMG activity was measured using reusable Ag/AgCl electrodes with a 2 mm contact area over corrugator and zygomaticus muscles on the right side of the face (van Boxtel, 2010). We included the zygomaticus (smiling) muscle to afford compatibility with previous studies addressing emotional valence. However, as Larsen et al. (2003) note, the zygomaticus does not reflect emotional valence in the same two-directional way that the corrugator does. The same study also showed that zygomaticus was less reliable as an indicator of valence in the case of linguistic stimuli. We would add to this that, especially in more complex environments such as our narrative stimuli, smiling activity may be difficult to interpret: smiles can be wry, sarcastic, and smirking as well as expressions of true positive feeling. We therefore focus on the corrugator, and report the zygomaticus data in Supplementary Figure 1 for reference. Raw EMG signals were recorded with a NeXus-10 MKII biosignal system (Mind Media) at a sampling rate of 2,048 Hz. After finishing this part of the experiment, electrodes were removed, participants moved to a laptop to fill out the questionnaires, and finally received their payment. Although, due to time-constraints, no comprehension questions were included, answers given in the exit-questionnaire indicated participants had in fact paid close attention to the stories.

### Data preparation and analysis

#### EMG data

The raw data were band-pass filtered between 20 and 500 Hz (48 dB/octave roll-off) and were additionally filtered with a notch filter at 50 Hz (see van Boxtel, 2010 for justification of filter parameters), followed by signal rectification and segmentation per narrative, all in BrainVision Analyzer 2. For each narrative, the 3,000 ms before story onset, consisting of the presentation of a neutral distractor image of a forest scene, were inspected for remaining artifacts. We selected maximally long continuous baseline epochs, with the requirement of a minimum of 500 ms of artifact-free signal for both muscles simultaneously. Trials were excluded if such a 500 ms baseline epoch could not be found (data loss of 0.50%).

Following baseline selection, data were exported to MatLab and then further segmented in two epochs of 5,000 ms, time-locked to the onsets of the character morality and critical event segments in each story. Next, the data for both the character morality segment and the critical event segment were divided into 50 consecutive 100 ms bins for optimal temporal resolution and reduction of random error, with the average EMG response in each bin expressed as a percentage of the pre-story baseline epoch (expressing responses as a percentage of baseline helps reduce random variance both within and between individuals; van Boxtel, 2010). Supplementary Figure 2 shows continuous average activation in 100 ms bins for an entire trial.

We performed a Mixed Models Linear Regression analysis (using SPSS version 24) for both critical segments. Rather than simply looking at average activation over the whole segment, we built a growth curve model that also captured linear, quadratic and cubic trends in the signal (Peck and Devore, 2008; Mirman, 2014; trend components were centered to avoid correlation between trend components). Using three trend components gives us two flex points and allows us to describe a response in some detail while retaining interpretability and avoiding over-fitting, as every extra flex point will make it easier to fit the data (Mirman, 2014). Models were fitted with 100-ms resolution, but for ease of comprehension, parameter estimates (e.g., a *b* for a linear slope) will be reported per second. Note that while we do not report effect size metrics, our parameter estimates reflect percentages and as such already give an indication of the size of the effect of a given predictor.

Rather than as one variable, we included separate trend components for each condition and added these iteratively. For each condition (moral & immoral for character manipulation and moral-pos/neg & immoral-pos/neg for critical event) we generated separate variables for linear, quadratic, and cubic trends. This allowed us to achieve maximal modeling flexibility without forcing the model to fit, for instance, a quadratic trend for all conditions when only some contained a significant quadratic component.

The model included subjects and items over lists as random intercepts, and random slopes for the trend components on the subject factor, but in the Results we only report (comparisons on the) linear component. We added predictors iteratively and used the −2LL chi-square test of model fit to assess whether each added factor improved the model (*p* < 0.05). Supplementary Information A contains the complete model summaries. The estimates of fixed effects reported in the results section belong to the final best-fit models.

#### Questionnaire data

For the transportability questionnaire, we calculated a basic total score over all questions ranging from 20 to 180 (cronbach's α in the current data set = 0.860). The AMES questionnaire contained three subscales each ranging from 1 to 5: affective empathy (cronbach's α in the current data set = 0.797), cognitive empathy (cronbach's α = 0.869), and sympathy (cronbach's α = 0.639). We thus calculated the averages for each subscale based on the original authors' validation study (Vossen et al., 2015).

To analyse the individual differences, we used the mixed models procedure again, but without the growth curve to avoid overcomplicating the analysis with four-way interactions. As the individual differences were of secondary interest for our main research question, the analysis we employed here was simplified. We immediately tested for the effect of each trait on average corrugator activation over the entire 5,000 ms of each of the manipulated segments, as well as the interaction with our conditions. Individual differences were entered as covariates in the fixed part of the model. We did not explore interactions between individual differences (e.g., between transportability and affective empathy) as we had no a priori reasons to do so.

## Results

### Character morality manipulation

**Figure 3:**
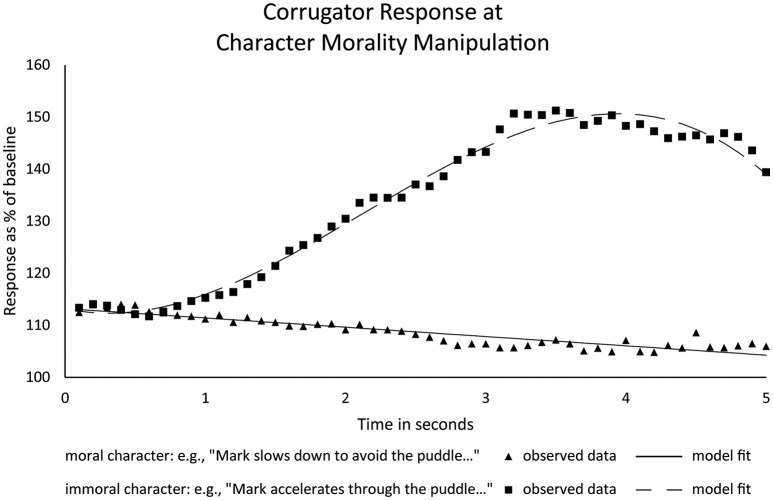
Observed averages of corrugator response during character morality segment, with growth curve model regression overlaid.

As can be seen in Figure 3, reading about moral and immoral actions elicited a clearly differential corrugator response^2^. For moral actions we saw a gradual and modest decrease in activity. In contrast, we found a rapid and substantial increase in corrugator activity starting within the first second in response to immoral actions of the main character. Statistical analysis corroborated these observations. First, the fixed effects revealed an effect of character morality, with immoral actions leading to higher activation than moral actions (difference *b* = 29.46, *t*_(26.42)_ = 13.02, *p* < 0.001, 95% CI of [25.00, 33.91])^3^. Furthermore, whereas moral actions induced a significant linear decrease in corrugator activity (*b* = −1.79, *t*_(59.99)_ = −4.02, *p* < 0.001, 95% CI [−2.69, −0.90]), the regression for immoral actions included a significant linear increase in corrugator activity (*b* = 15.14, *t*_(59.97)_ = 4.55, *p* < 0.001, 95 % CI [8.48, 21.80]), with both linear trends also differing significantly from each other (*p* < 0.001; all slope estimates per second). This is in line with our predictions that moral actions would elicit positive affect and immoral actions would elicit negative affect. It also suggests that the stage was effectively set for our subsequent critical event manipulation. For a complete report of the results, see Supplementary Information A.

### Critical event manipulation

**Figure 4:**
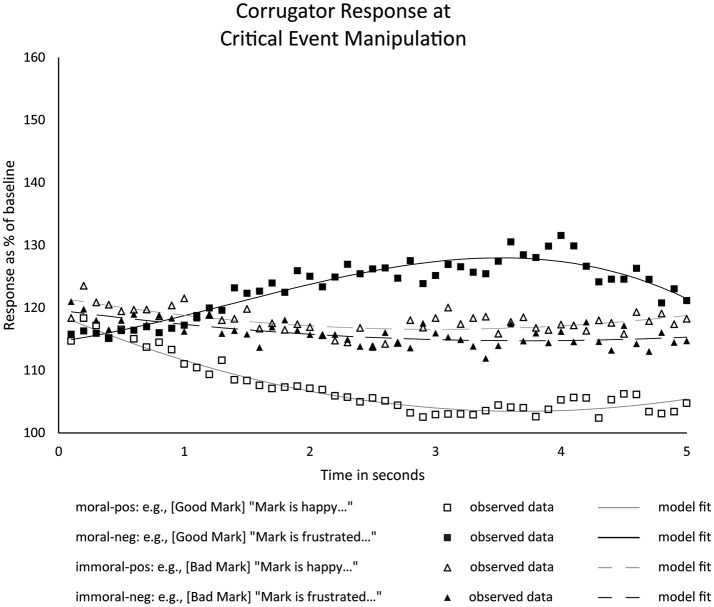
Observed averages of corrugator response to critical events befalling moral and immoral characters, with growth curve model regression overlaid.

Figure 4 below shows the results for the critical event manipulation: something positive or negative befalling the character. Our predictions concerned the effect of the character morality on the valence of the corrugator response, in relation to the valence of the event. We will therefore discuss the critical events separately for moral and immoral characters.

#### Moral characters

The corrugator response to critical events happening to moral characters revealed a clear differential pattern for positive and negative events (see Figure 4, squares and solid lines), with negative events eliciting an increase in corrugator activity, and positive events leading to a decrease in activity. This pattern was corroborated by statistical analysis. For moral characters, negative events elicited significantly stronger mean corrugator activation than positive events (moral-negative - moral-positive difference: *b* = 21.11, *t*_(272.33)_ = 8.19, *p* < 0.001, 95% CI of [16.03, 26.18]), and also resulted in clearly different temporal developments. In particular, whereas the model regression for moral-negative contained a linear *increase* in activation (*b* = 3.60, *t*_(95.84)_ = 3.20, *p* = 0.01, 95% CI of [1.37, 5.84]), moral-positive conditions led to a linear *decrease* in activation (*b* = −2.58, *t*_(59.74)_ = −4.54, *p* < 0.001, 95% CI [−3.71, −1.44]). Note, all slope estimates are reported *per second*. A pairwise comparison of these linear trends also revealed they differed significantly at the *p* < 0.001 level.

Although these different corrugator results reflect a difference in valence of the critical event, the data at this point do not allow us to say whether the corrugator response here reflects language-driven simulation, moral evaluation, or a mixture of the two. This is because in the case of moral characters, simulation and evaluation predict the same valence for the corrugator response. To illustrate, language describing a negative event befalling a moral character would involve simulation of negative concepts or negative character emotions in the situation model and as such lead to increased corrugator activity. By the same token however, the evaluation of a negative event befalling a good character as *unfair* would also lead to increased corrugator activity. Our design included the immoral character conditions to pull these potential drivers of the corrugator response apart.

#### Immoral characters

The corrugator response for immoral-positive and immoral-negative conditions (see Figure 4, triangles and dashed lines) presented a very different picture from that for the moral characters. At first glance, the immoral conditions did not show a clear valenced response at all. The statistical model confirmed this impression. With immoral characters, mean corrugator activity did not significantly depend on whether a positive or negative event occurred (immoral-positive - immoral-negative difference: *b* = 1.37, *t*_(272.40)_ = 0.53, *p* = 0.60, 95% CI [−3.71, 6.44]). Furthermore, although including linear and quadratic components for both positive and negative events befalling characters significantly improved the model, none of the linear and quadratic estimates themselves significantly different from zero (see Supplementary Information A). In all, the statistics indicate a flat-line corrugator response in both cases and no significant difference between the two.

These results do not sit well with the *simulation-only* account (which predicted similar corrugator effects of negative vs. positive events regardless of protagonist status), nor with the *evaluation-blocks-simulation* account (which predicted that the pattern of corrugator activation would track fairness, and hence flip for bad protagonists, relative to good protagonists). Because the results cannot easily be explained in either of these simple models, they suggest that some version of the *multiple-drivers* account, where both language-driven simulation and moral evaluation exerted control over the corrugator, is appropriate here. We will explore this further in the Discussion section.

### Individual differences

We analyzed the individual differences by entering each personality trait as a continuous covariate in the fixed part of a simplified model which included morality and critical event, but excluded linear, quadratic and cubic trends (see section Methods). The personality traits included Cognitive Empathy (*M* = 3.54, range = 2.17–4.83) Affective Empathy (*M* = 3.01, range = 1.60–4.00) Sympathy (*M* = 3.86, range = 2.5–4.75) and Transportability (*M* = 73.19, range = 39–132). Our models revealed that only Affective Empathy and Transportability significantly modulated corrugator activity (see Supplementary Information B).

At the character morality segment, Affective Empathy scores only had an effect on corrugator activity elicited by immoral actions (*b* = 16.16, *t*_(61.74)_ = 3.46, *p* < 0.001, 95% CI [6.84, 25.48]), with higher Affective Empathy scores associated with more negative affect. At the same segment, Transportability also modulated the corrugator responses elicited by immoral actions only (*b* = −0.55, *t*_(62.05)_ = −3.42, *p* < 0.001, 95% CI [−0.87, −0.23]), with higher Transportability scores associated with *less* negative affect. Affective Empathy and Transportation thus seemed to have the opposite effect on frowning activity during immoral actions. Because Affective Empathy and Transportation scores themselves were negatively correlated [*r*_(58)_ = −0.17, *p*_(*two*−*tailed*)_ < 0.001], their impact on corrugator activity may not be independent. Corrugator activity to moral actions did not show a significant effect of either Affective Empathy scores (*p* = 0.40) or Transportation scores (*p* = 0.54).

At the critical event segment, we found that in the moral-negative condition (bad things happening to good people) higher Affective Empathy scores once again resulted in more corrugator activity (*b* = 9.17, *t*_(62.72)_ = 2.36, *p* = 0.02, 95% CI [1.39, 16.95]), indicating that those higher in Affective Empathy clearly exhibited more negative affect in response to the character experiencing a negative emotion. Furthermore, we found the same relationship between corrugator activity and Affective Empathy for the immoral-positive condition (*b* = 8.61, *t*_(62.78)_ = 2.21, *p* = 0.03, 95% CI [0.82, 16.39]). As both conditions can be considered to involve an evaluation in terms of *unfairness*, a parsimonious explanation might be that those predisposed to feel what others feel display more negative affect particularly in cases of unfairness, an account that can also explain the Affective Empathy effect on corrugator responding to the first immoral action of the character. At the critical event segment, Transportation scores did not co-vary with corrugator activity in any of the conditions (see Supplementary Information B).

## Discussion

Using a recent model of affective language comprehension (Van Berkum, 2018a,b) as our guide, this study pitted language-driven simulation against moral evaluation using narratives containing a protagonist manipulated to be moral or immoral. We used EMG to measure the response of the corrugator muscle as an indicator of these two processes. For the character morality manipulation (e.g., “Mark slows down/accelerates…”), we expected that the corrugator response would clearly reflect moral valence. This prediction was borne out by our results: relative to a pre-story baseline, participants frowned more at immoral actions and less at moral actions, with the difference between the two emerging relatively rapidly, in less than a second after presentation of the critical sentence. This adds to existing evidence that facial EMG recordings in general, and that of the corrugator in particular, can help track responses to affectively loaded language (e.g., Foroni and Semin, 2009, 2013; Glenberg et al., 2009; Niedenthal et al., 2009), and extends that evidence to a new domain, the processing of *morally* loaded language (e.g., Van Berkum et al., 2009; Leuthold et al., 2015, for EEG indications of very rapid processing of such language).

The character morality manipulation was crucial to untangle the two possible drivers behind the corrugator response at the *subsequent* critical event (e.g., “Mark was happy/frustrated when…”). Here, the presence of immoral characters led to conflicting valence predictions depending on whether language-driven simulation or fairness-based moral evaluation drove the corrugator response, both in the immoral-negative condition (a bad person befalls something bad, i.e., negative for the character but fair in the eyes of the reader) and the immoral-positive condition (a bad person befalls something good, i.e., positive for the character but unfair in the eyes of the reader). We considered three accounts of how corrugator activity might reflect this conflict during the critical event manipulation: (1) the corrugator response might simply track the valence of language-driven simulation of the event (*simulation-only* account), (2) the corrugator response would reflect only fairness-based moral evaluation (*evaluation-blocks-simulation* account), or (3) language-driven simulation and moral evaluation would both drive the corrugator response (*multiple-drivers* account).

Because the observed pattern of corrugator activity radically depends on the moral status of the protagonist, our data clearly refute a simple *simulation-only* account, according to which corrugator responses to phrases such as “Mark was frustrated/happy” merely reflect generic situation-model building (imagining the protagonist as frustrated or happy) and/or simulating lexical-conceptual meaning (“frustrated”, “happy”) in the service of such construction. We already considered this account rather unlikely, in part because socially important uses of narrative in human exchanges, such as gossip, can only work if moral evaluation does *not* shut down when people process language (Dunbar, 2004). Of course, when reading fictional narratives in the lab, real social relations are not at stake and evaluative responses therefore might well be attenuated. Given that moral evaluation is to a large extent automatic (Greene, 2014), however, it is unlikely that it would be eliminated in the lab. Our corrugator data at the critical event confirm this idea of automatic moral evaluation, as does the large and rapid corrugator response to a moral transgression earlier in the narrative.

Our results also refute the more plausible *evaluation-blocks-simulation* account, which had predicted the corrugator responses to critical events to “flip” as a function of whether the protagonist had just behaved morally (stronger corrugator activity for negative events than positive events) or immorally (stronger corrugator activity for positive events than negative events). This is not what our data show: whereas negative events lead to considerably more corrugator activity than positive events when those events befell a morally laudable person, there was no difference in corrugator activity between positive and negative events befalling immoral characters. The results displayed in Figure 4 are thus best accounted for in terms of a *multiple-drivers* account, where both language-driven simulation of events (as part of lexical retrieval and/or situation model construction) *and* the evaluation of those events (i.e., the reader's own emotions) simultaneously recruit the neural systems driving the corrugator, at least with the materials studied here.

How those two drivers interact *exactly* is as yet an open question, constrained neither by our data nor by the ALC model that we used to formulate predictions. One possibility is that in the case of immoral characters, the corrugator activations associated with language-driven simulation and moral evaluation cancel each other out. In this version of the multiple-drivers account, the corrugator increase due to, say, simulating a frustrated protagonist and/or retrieving the associated lexical semantics would then need to be leveled out by a corrugator *de*crease related to the positive affect associated with evaluating this state of affairs as *fair* (e.g., a “*serves-you-right”* sense of justice, Schadenfreude) in case this protagonist previously behaved immorally. Likewise, with an immoral protagonist, the corrugator decrease due to simulating his or her happiness and/or retrieving the associated lexical semantics would then need to be leveled out by a corrugator *in*crease related to the negative affect associated with evaluating this particular outcome as *unfair* (a sense of moral indignation, anger, etc.)^4^.

Corrugator EMG has also been linked to ease of processing or mental effort (e.g., Cacioppo et al., 1985; Cohen et al., 1992; Topolinski et al., 2009) where enhanced corrugator activity is indicative of increased effort and less fluency in processing. At first glance, this may seem to be a potential confound for our results. However, the immoral conditions are arguably the most complicated because evaluation in these cases involves a reassessment of the valence of the event in light of the character's moral status. Yet, for these conditions we do not find any phasic increase in corrugator actvity at the critical event. In fact, the only time corrugator EMG increases during the critical event is when good characters experience a negative event, arguably a fairly uncomplicated case. As such, complexity or disfluency in processing does not offer a parsimonious explanation for the patterns we found during the critical event segment.

An alternative account that we cannot as yet fully rule out relates to the concept of identification. Identification with characters is often assumed to involve the reader taking on the character's goals and values as their own and experiencing the emotions of the character (Oatley, 1995). While the ALC model does not discuss mechanisms of identification, they could in the context of that model be reframed in terms of *selective*, context-dependent simulation at the level of the situation model, i.e. of simulating the emotions of certain protagonists, but not those of others. There is evidence for a connection between character likeability and identification (Tian and Hoffner, 2010; Chory, 2013) and reduced self-reported experience of positive and negative emotions in response to characters that readers identified with less (Hoeken and Sinkeldam, 2014). More generally, the emotional charge of the context appears to affect both what and how much is simulated (Samur et al., 2015). If readers only simulate the emotions of likable characters and *not* those of disliked characters, a result such as in Figure 4 is conceivable. To explain our corrugator findings for immoral characters with this “selective simulation” account *alone*, however, one would also need to assume that readers do not morally evaluate what they read or hear, at least not in the case of events befalling immoral characters. With morally loaded narrative, and for reasons discussed before, we deem such absence of moral evaluation highly unlikely. Also, more generally, we find it unlikely that motor control programs that evolved to express one's own emotion are merely used to simulate other people's emotions, and not used to express ones own emotion over rather eventful social matters. If this were the case, people would for example not be able to facially express, to each other, their emotional alignment over a piece of gossip, or some other narrative about what happened to them or others.

As for follow-up experiments on the interplay between simulation and evaluation, note that the ALC model makes explicit two different potential loci for affective simulation with language comprehension, which can in principle be independently manipulated via, e.g., negation (“Mark was not furious”). Crossing such a simulation-type manipulation with evaluation might help in teasing out which precise aspect(s) of affective simulation co-occur with affective evaluation. One possible scenario is that whereas simulation that is part of conceptual retrieval is obligatory and as such not blocked by evaluation, the simulation involved in situation model construction might be tuned down or blocked altogether (cf. Zwaan, 2014).

It would also be useful to obtain more precise evidence on how the corrugator response unfolds relative to crucial words in the unfolding sentence. Our critical sentences were presented as a whole in our current study, so it is not exactly known when critical words like *frustrated* or *happy* were read by the participants. More precise time-locking to words like *happy* or *frustrated* might thus reveal an initial purely simulation-driven corrugator response, rapidly followed by an evaluative or mixed response. Furthermore, our critical events can be said to consist of two components: an adjective which signals the valence of the emotional state of the character (e.g., *happy* vs. *frustrated*), and a clause describing the reason for this state (e.g., *when there isn't a petrol station in sight and…*). Presenting the latter part of the stimulus a little later than the adjective would allow us to observe the separate EMG response to either. A more precisely time-locked version of the same experiment with a more fine-grained control of when specific information becomes available might thus help to differentiate the respective contributions of simulation and evaluation to the unfolding corrugator EMG signal.

The effects of Affective Empathy suggest that the corrugator response to morally loaded narrative is also subject to some individual variation. At the character morality manipulation, readers with a higher Affective Empathy score frowned more at immoral actions, and at the critical event manipulation, those readers frowned more in response to bad things happening to good characters as well as to good things happening to bad characters. A reasonable post hoc interpretation is that people who are by habit or constitution more predisposed to feel what others feel are also particularly responsive to *un*fairness, rather than fairness. Furthermore, we found that readers with higher Transportability scores responded *less* to descriptions of immoral actions than those with lower scores. This could be taken to suggest that moral judgment of narratives decreases when a reader is more immersed in the narrative, perhaps because of a shift in the balance between evaluation and simulation. As Transportability correlated negatively with Affective Empathy in our sample, these effects might not be independent of each other. While these results provide food for thought, the evidence is correlational, and must as such be approached with great caution.

In all, and independent of which precise variant of the multiple-drivers account will ultimately account for them, our results suggest that moral evaluation has powerful effects on corrugator activity during narrative language processing: sentences containing affective information such as “Mark is furious when…” and “Mark is happy when…” generate quite different corrugator EMG responses as a function of whether the protagonist has just displayed morally good or objectionable behavior. We take this result to reflect the reader's direct and rapid moral evaluation of, and associated emotional response to, what is being narrated, both when reading about downright moral or immoral behavior, and when reading about events that befall the characters at hand. The implication is that corrugator activity during language processing does not merely reflect simulation of the protagonist's emotion (e.g., Mark being frustrated) and/or of lexical-semantic meaning (e.g., retrieval of the meaning of *frustrated*). The fact that people have emotions about other people's emotions co-determines how their corrugator responds as they read a story about those other people.

Although the traces of affect picked up via facial EMG over the corrugator may often not be visible in the face (Tassinary and Cacioppo, 1992), corrugator EMG recordings show us that readers quite literally frown upon descriptions of characters behaving immorally and that the moral status of characters drastically influences corrugator activity during later affectively salient passages. This result highlights the importance of unpacking coarse notions of affective meaning in language processing research into components that reflect not only simulation but also evaluation. In line with a central tenet of the *Affective Language Comprehension* model, our corrugator EMG results here also call for a re-evaluation of the “simple” interpretation of corrugator EMG (and other affect-related facial muscles) and other peripheral physiological measures as unequivocal indicators of *simulation* in affective language processing. Further exploration is needed of how such measures behave in a richer and more ecologically valid language processing arena, such as narrative. Such work would benefit the field by refining our understanding of the role of simulation processes within a framework of grounded cognition in general and language comprehension in particular.

## Author contributions

B'tH, MS, and JvB designed the study; B'tH conducted the study; B'tH and MS analyzed the results, B'tH, MS, and JvB wrote the paper; TvB provided specific EMG expertise for study design and data analysis.

### Conflict of interest statement

The authors declare that the research was conducted in the absence of any commercial or financial relationships that could be construed as a potential conflict of interest.

## Footnotes

^1^We prefer the AMES over alternative empathy questionnaires (such as the Interpersonal Reactivity Index, Davis, 1983) because it separates cognitive and affective empathy in a more principled way (see Vossen et al., 2015, for discussion). Although originally targeting adolescents, the items that make up the AMES scales are appropriate for our relative young (student population) age group, and, importantly, scoring does not involve norming scores relative to an adolescent score distribution (p. 8).

^2^The corrugator responses in Figure 3 start slightly above the 100% baseline level. We suspect this reflects the additional effort demanded by reading the preceding introduction segment of the narratives, in comparison with passive viewing of the preceding neutral image (p. 13).

^3^Because our growth curve analysis used *centered* time components, the intercept in our analyses represents the average activation over the entire time window, rather than the point where the curves leave the Y-axis. Furthermore, whereas the reported intercepts are based on fixed effects in the final model only, the fitted line in Figure 3 includes both the fixed effects and the random effects from the model (p. 13).

^4^Although such exact cancelation may feel somewhat “coincidental,” we note that this outcome is as likely as any specific *partial* cancelation, such as when the impact of evaluation is twice that of the counteracting impact of simulation (p. 20).

## Supplementary material

Supplementary Figure 1 Observed averages of zygomaticus response during character morality (above) and critical events befalling moral and immoral characters; with growth curves overlaid.

Supplementary Figure 2 Total observed averages whole trial for each condition.
