# Supplementary figures and images for: Emotion in Stories: Facial EMG Evidence for Both Mental Simulation and Moral Evaluation
Source: Front Psychol. 2018 Apr 30;9:613. doi: 10.3389/fpsyg.2018.00613 (PMC5937160; doi:10.3389/fpsyg.2018.00613)

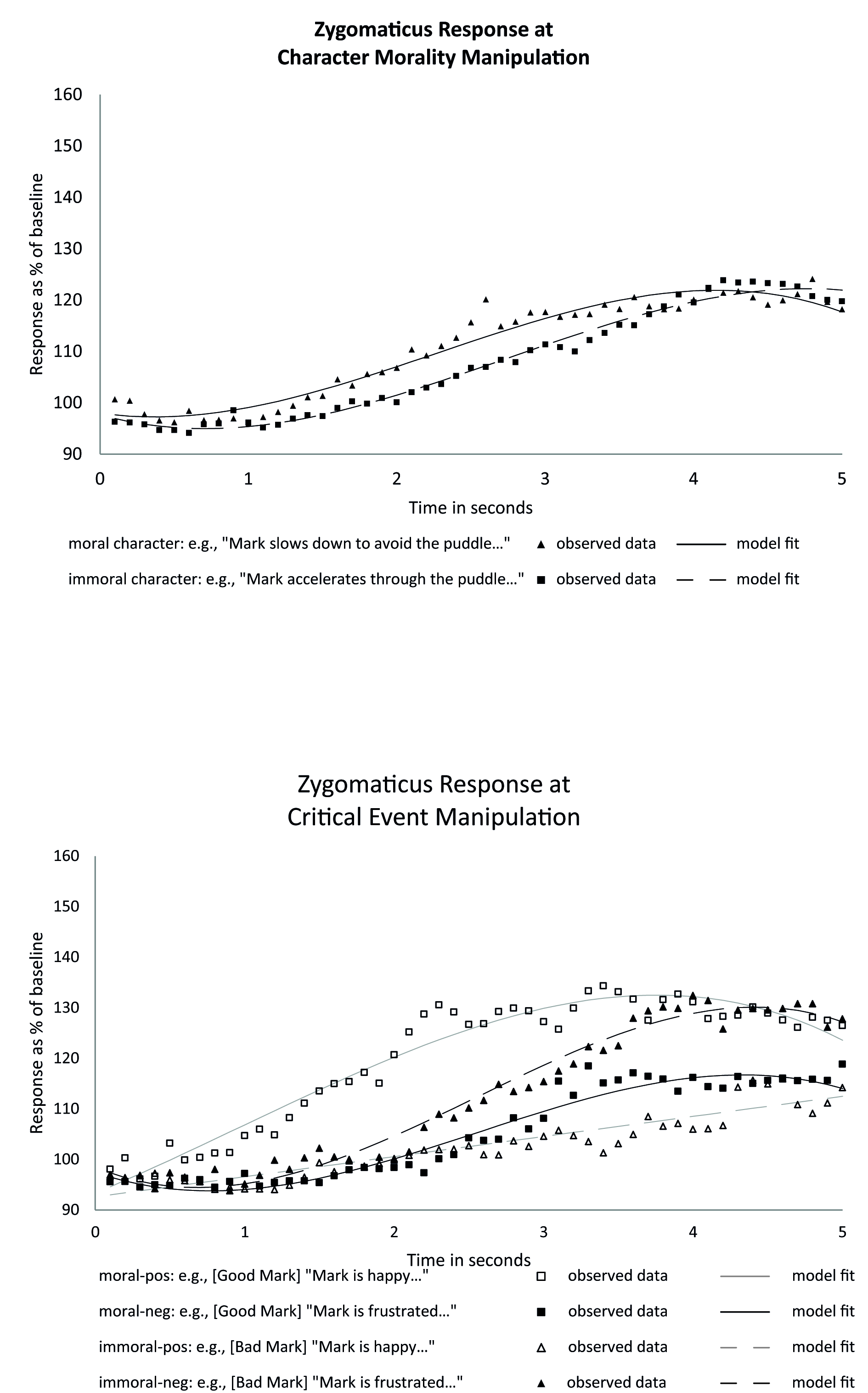

Supplement: Supplementary Figure 1 — Observed averages of zygomaticus response during character morality (above) and critical events befalling moral and immoral characters; with growth curves overlaid. [file Image_1.TIF]

### Observed Averages Corrugator Whole Trial

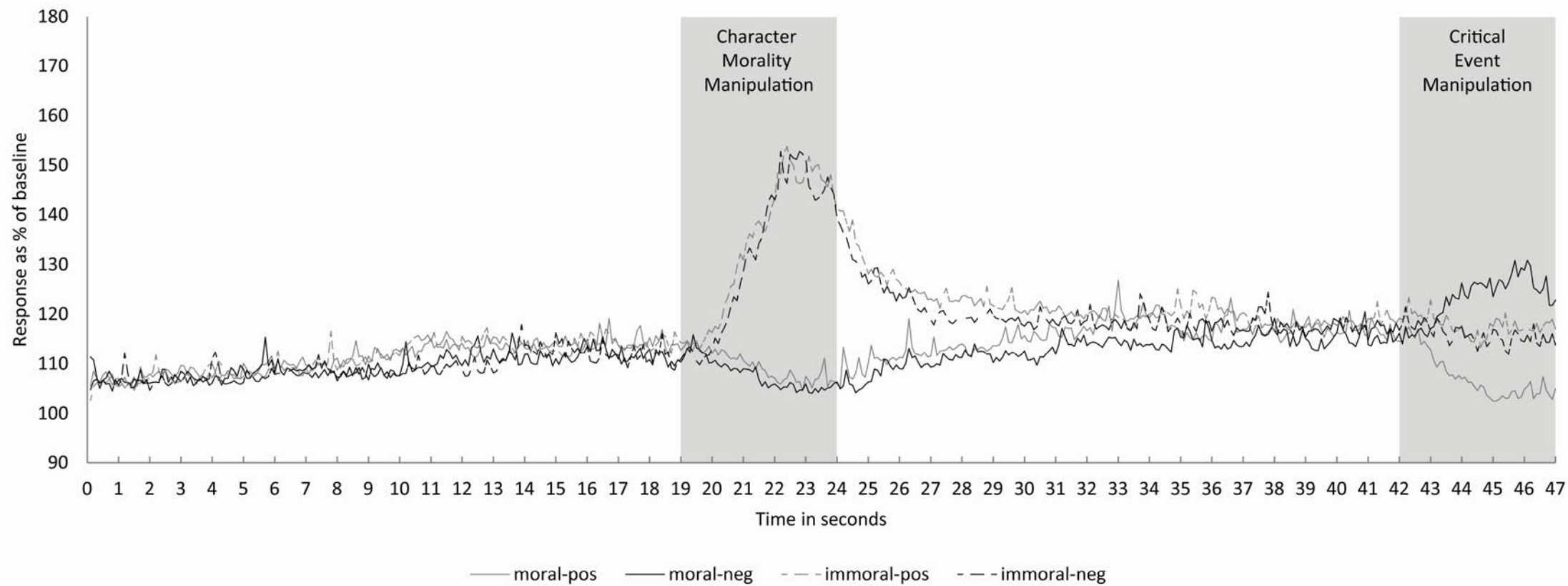

Supplement: Supplementary Figure 2 — Total observed averages whole trial for each condition. [file Image_2.PDF]
