# Supplementary material for: Emotion in Stories: Facial EMG Evidence for Both Mental Simulation and Moral Evaluation
Source: Front Psychol. 2018 Apr 30;9:613. doi: 10.3389/fpsyg.2018.00613 (PMC5937160; doi:10.3389/fpsyg.2018.00613)
Supplement: Supplementary file 3 [file Table_1.PDF]

Supplementary Information A - Model Summaries and Estimates

	Character Morality Corrugator	total cases	cases after baseline rejection	data loss
		191900	190950	0,50%

Iterative model report for Character Morality Manipulation. Each line reports the assessment of improved model fit after adding a single predictor.

Nr.	-2 LL	nr of parameters	p model fit (chisquare distribution)	model comparision	predictor added	action
Model 0	2297839,064	2			empty model	-
Model 1	2288296,555	3	0,000	better	Subject Random Intercept	keep
Model 2	2279588,985	4	0,000	better	Item Random Intercept	keep
Model 3	2279517,602	5	0,000	better	Moral Linear Time Fixed	keep
Model 4	2277709,547	6	0,000	better	Immoral Linear Time Fixed	keep
Model 5	2277553,13	8	0,000	better	Moral Linear Time Random Slope (Subject UN)	keep
Model 6	2272924,031	11	0,000	better	Immoral Linear Time Random Slope (Subject UN)	keep
Model 7	2272922,516	12	0,218	not better	Moral Quadratic Time Fixed	remove
Model 8	2272791,796	12	0,000	better	Immoral Quadratic Time Fixed	keep
Model 9	2272789,978	13	0,178	not better	Moral Cubic Fixed	remove
Model 10	2272633,675	13	0,000	better	Immoral Cubic Time Fixed	keep
Model 11	2272502,391	14	0,000	better	Character Morality Conditie	keep
Model 12	2270884,847	18	0,000	better	Immoral Quadratic Time Random Slope (Subject UN)	keep
Model 13	2270262,398	23	0,000	better	Immoral Cubic Time Random Slope (Subject UN)	keep
Model 14	2270261,652	24	0,388	not better	Partcipant Gender	remove

Supplementary Information A - Model Summaries and Estimates

Estimates of Fixed Effects Character Morality[a]

Parameter	Estimate	Std. Error	df	t	Sig.	95% Confidence Interval	
						Lower Bound	Upper Bound
Linear Moral	-1,7949603	0,446352979	59,98608	-4,02139201	0,0002	-2,687803455	-0,902117152
Linear Immoral	15,1401722	3,329628313	59,96913	4,54710581	0,0000	8,479853458	21,80049102
Quadratic Immoral	-2,0202699	0,697804456	62,42766	-2,89518057	0,0052	-3,414971073	-0,625568725
Cubic Immoral	-1,6228965	0,448368177	59,87714	-3,61956229	0,0006	-2,519804284	-0,725988806
Immoral	138,172822	3,007079108	108,9822	45,9491808	0,0000	132,2128776	144,1327655
Moral	108,716885	2,988543785	106,3199	36,3778791	0,0000	102,7920117	114,6417575

a. Dependent Variable: Character Morality Corrugator Response.

Pairwise Comparisons Main Effect of Character Morality[a]

(I) likeability manipulation		Mean Differenc	Std. Error	df	Sig.[c]	95% Confidence Interval for Difference[c]	
						Lower Bound	Upper Bound
moral	immoral	-29,456*	2,261685	264,421122	0,0000	-33,9091399	-25,00273402

Based on estimated marginal means

*. The mean difference is significant at the ,05 level.

a. Dependent Variable: Character Morality Corrugator Response.

c. Adjustment for multiple comparisons: Bonferroni.

Pairwise Comparison between Linear Estimates Moral & Immoral[a,b]

Contrast	Estimate	Std. Error	df	Test Value	t	Sig.	95% Confidence Interval	
							Lower Bound	Upper Bound
Linear Moral vs. Linear Immoral	-16,928384	3,22990471	62,12162	0	-5,24114	0,0000	-23,38462049	-10,47214833

a. linear moral vs immoral

b. Dependent Variable: Character Morality Corrugator Response.

Supplementary Information A - Model Summaries and Estimates

	total cases	cases after baseline rejection	data loss
Critical Event Corrugator	191900	190950	0,50%

Iterative model report for Critical Event Manipulation. Each line reports the assessment of improved model fit after adding a single predictor.

Nr.	-2 LL	nr of parameters	p model fit (chisquare distribution)	model comparision	predictor added	action
Model 0	2194785,534	2			empty model	
Model 1	2183526,383	3	0,000	better	Subject Random Intercept	keep
Model 2	2176247,842	4	0,000	better	Item Random Intercept	keep
Model 3	2176121,731	5	0,000	better	Linear Time Moral Positive Fixed	keep
Model 4	2176116,686	6	0,025	better	Linear Time Immoral Positive Fixed	keep
Model 5	2176103,552	7	0,000	better	Linear Time Immoral Negative Fixed	keep
Model 6	2176011,053	8	0,000	better	Linear Time Moral Negative Fixed	keep
Model 7	2175796,515	10	0,000	better	Linear Time Moral Positive Random Slope (Subject UN)	keep
Model 8	2175386,242	13	0,000	better	Linear Time Immoral Positive Random Slope (Subject UN)	keep
Model 9	2174903,38	17	0,000	better	Linear Time Immoral Negative Random Slope (Subject UN)	keep
Model 10	2173945,4	22	0,000	better	Linear Time Moral Negative Random Slope (Subject UN)	keep
Model 11	2173915,017	23	0,000	better	Quadratic Time Moral Positive Fixed	keep
Model 12	2173903,89	24	0,001	better	Quadratic Time Immoral Positive Fixed	keep
Model 13	2173899,692	25	0,040	better	Quadratic Time Immoral Negative Fixed	keep
Model 14	2173855,657	26	0,000	better	Quadratic Time Moral Negative Fixed	keep
Model 15	2172900,165	32	0,000	better	Quadratic Time Moral Positive Random (Subject UN)	keep
Model 16	2172408,175	39	0,000	better	Quadratic Time Immoral Positive Random (Subject UN)	keep
Model 17	2171607,151	47	0,000	better	Quadratic Time Immoral Negative Random (Subject UN)	keep
Model 18	2171061,953	56	0,000	better	Quadratic Moral Negative Random (Subject UN)	keep
Model 19	2171061,828	57	0,724	not better	Cubic Time Moral Positive Fixed	remove
Model 20	2171061,634	57	0,572	not better	Cubic Time Immoral Positive Fixed	remove
Model 21	2171061,656	57	0,586	not better	Cubic Time Immoral Negative Fixed	remove
Model 22	2171054,394	57	0,006	better	Cubic Time Moral Negative Fixed	keep
Model 23	0	0	0,000	no convergence	Cubic Time Moral Negative Random (Subject UN)	remove
Model 24	2171054,349	58	0,832	not better	Character Morality	keep for interaction
Model 25	2171029,814	59	0,000	better	Event Valence	keep
Model 26	2170994,271	60	0,000	better	Character Morality * Event Valence	keep
Model 27	2170993,277	61	0,319	not better	Participant Gender	remove

Supplementary Information A - Model Summaries and Estimates

Estimates of Fixed Effects Critical Event[a]

Parameter	Estimate	Std. Error	df	t	Sig.	95% Confidence Interval	
						Lower Bound	Upper Bound
Linear Moral Pos	-2,57790482	0,567606584	59,74204	-4,54171057	0,0000	-3,713387875	-1,442421766
Linear Immoral Pos	-0,490665694	0,714347637	59,89735	-0,68687243	0,4948	-1,919624083	0,938292696
Linear Immoral Neg	-0,849905946	0,764431669	59,99941	-1,11181415	0,2707	-2,378997259	0,679185368
Linear Moral Neg	3,603092945	1,126370925	95,84188	3,19885116	0,0019	1,367217253	5,838968636
Quadratic Moral Pos	1,109009004	0,567521612	64,89307	1,95412647	0,0550	-0,024445327	2,242463335
Quadratic Immoral Pos	0,554552174	0,439062305	68,25068	1,26303754	0,2109	-0,321524751	1,430629099
Quadratic Immoral Neg	0,334638614	0,524259957	65,81772	0,63830664	0,5255	-0,712134274	1,381411503
Quadratic Moral Neg	-1,31861801	0,484270577	66,47937	-2,72289516	0,0083	-2,285365298	-0,351870722
Cubic Moral Neg	-0,377622256	0,137347546	190155,6	-2,74939208	0,0060	-0,646820127	-0,108424384
Immoral Neg	115,3307653	3,194548204	122,1733	36,1023713	0,0000	109,0069279	121,6546027
Immoral Pos	116,6956777	3,194550926	122,1737	36,5296032	0,0000	110,3718352	123,0195203
Moral Neg	126,1326559	3,194385862	122,1485	39,4857295	0,0000	119,809127	132,4561848
Moral Pos	105,0267815	3,19459117	122,1799	32,8764389	0,0000	98,70286248	111,3507005

a. Dependent Variable: Critical Event Corrugator Response.

Pairwise Comparisons Character Morality*EventValence[a]

(I) reference like-pos		Mean Difference (I-J)	Std. Error	df	Sig.[c]	95% Confidence Interval for Difference[c]	
						Lower Bound	Upper Bound
immoralneg	immoralpos	-1,3650	2,5780	272,3950	1,0000	-8,2170	5,4870
	moralneg	-10,8020	2,5780	272,3110	0,0000	-17,6530	-3,9500
	moralpos	10,3040	2,5780	272,4140	0,0000	3,4520	17,1560
immoralpos	moralneg	-9,4370	2,5780	272,3130	0,0020	-16,2890	-2,5850
	moralpos	11,6690	2,5780	272,4180	0,0000	4,8170	18,5210
moralneg	moralpos	21,1060	2,5780	272,3330	0,0000	14,2540	27,9580

Based on estimated marginal means

*. The mean difference is significant at the ,05 level.

a. Dependent Variable: Critical Event Corrugator Response.

c. Adjustment for multiple comparisons: Bonferroni.

Pairwise Comparison between Linear Moral Pos & Linear Moral Neg[a,b]

Contrast	Estimate	Std. Error	df	Test Value	t	Sig.	95% Confidence Interval	
							Lower Bound	Upper Bound
Linear Moral Pos vs. Linear Moral Neg	-6,18159973	1,261746038	136,6626	0	-4,899242	2,67953E-06	-8,676670622	-3,686528837

a. linear mpos-mneg

b. Dependent Variable: Critical Event Corrugator Response.
