# Supplementary material for: Emotion in Stories: Facial EMG Evidence for Both Mental Simulation and Moral Evaluation
Source: Front Psychol. 2018 Apr 30;9:613. doi: 10.3389/fpsyg.2018.00613 (PMC5937160; doi:10.3389/fpsyg.2018.00613)
Supplement: Supplementary file 4 [file Table_2.pdf]

Supplementary Information B: Parameter Estimates of Individual Differences on Corrugator Response at Character Morality Segment

Highlighted are the *p* values for the effect of each individual differences measure on the average corrugator response for every condition

Estimates of Fixed Effects Character: Cognitive Empathy*Moral Character[a]

Parameter	Estimate	Std. Error	df	t	Sig.	95% Confidence Interval	
						Lower Bound	Upper Bound
Intercept	99,42435734	18,75019868	61,6981187	5,302576203	0,0000	61,93959589	136,9091188
Immoral Character	24,66045629	3,636486779	1755,765269	6,781395833	0,0000	17,52815647	31,79275611
Moral Character	0b	0					
Cognitive Empathy	2,631145015	5,221469595	60,88962041	0,503908903	0,6161	-7,810211539	13,07250157
Immoral Character * Cognitve Empathy	0,156494691	0,812115889	190266,8463	0,19269995	0,8472	-1,435232823	1,748222206
Moral Character * Cognitive Empathy	0b	0					

a. Dependent Variable: Character Morality Corrugator Response.
b. This parameter is set to zero because it is redundant.

Estimates of Fixed Effects Character: Cognitive Empathy*Immoral Character[a]

Parameter	Estimate	Std. Error	df	t	Sig.	95% Confidence Interval	
						Lower Bound	Upper Bound
Intercept	124,0848136	18,74995401	61,69497505	6,617872955	0,0000	86,60050306	161,5691241
Moral Character	-24,66045628	3,636486779	1755,765269	-6,781395831	0,0000	-31,7927561	-17,52815646
Immoral Character	0b	0					
Cognitive Empathy	2,787639727	5,221399635	60,88645077	0,533887448	0,5954	-7,653587942	13,2288674
Moral Character * Cognitive Empathy	-0,156494694	0,812115889	190266,8463	-0,192699953	0,8472	-1,748222208	1,435232821
Immoral Character * Cognitive Empathy	0b	0					

a. Dependent Variable: Character Morality Corrugator Response.
b. This parameter is set to zero because it is redundant.

Estimates of Fixed Effects Character: Sympathy*Moral Character[a]

Parameter	Estimate	Std. Error	df	t	Sig.	95% Confidence Interval	
						Lower Bound	Upper Bound
Intercept	119,0502739	21,89964791	60,83526086	5,436172966	0,0000	75,25682306	162,8437248
Immoral Character	7,634370578	4,002442522	2573,351893	1,907427911	0,0566	-0,213964015	15,48270517
Moral Character	0b	0					
Sympathy	-2,671023698	5,606233993	60,23832209	-0,47643814	0,6355	-13,88424889	8,54220149
Immoral Character * Sympathy	4,549460224	0,859961685	190864,5533	5,290305722	0,0000	2,863956138	6,23496431
Moral Character * Sympathy	0b	0					

a. Dependent Variable: Character Morality Corrugator Response.
b. This parameter is set to zero because it is redundant.

Estimates of Fixed Effects Character: Sympathy*Immoral Character[a]

Parameter	Estimate	Std. Error	df	t	Sig.	95% Confidence Interval	
						Lower Bound	Upper Bound
Intercept	126,6846445	21,8996125	60,83488945	5,78478932	0,0000	82,89125902	170,47803
Moral Character	-7,634370579	4,002442522	2573,351893	-1,907427911	0,0566	-15,48270517	0,213964015
Immoral Character	0b	0					
Sympathy	1,878436526	5,606244417	60,2387996	0,335061475	0,7387	-9,334807691	13,09168074
Moral Character * Sympathy	-4,549460223	0,859961685	190864,5533	-5,290305721	0,0000	-6,234964309	-2,863956138
Immoral Character * Sympathy	0b	0					

a. Dependent Variable: Character Morality Corrugator Response.
b. This parameter is set to zero because it is redundant.

Supplementary Information B: Parameter Estimates of Individual Differences on Corrugator Response at Character Morality Segment

Highlighted are the *p* values for the effect of each individual differences measure on the average corrugator response for every condition

Estimates of Fixed Effects Character: Affective Empathy*Moral Character[a]

Parameter	Estimate	Std. Error	df	t	Sig.	95% Confidence Interval	
						Lower Bound	Upper Bound
Intercept	96,78584327	14,36809853	63,09973879	6,736162272	0,0000	68,07437761	125,4973089
Immoral Character	-11,43920512	3,183989055	1066,358317	-3,592727526	0,0003	-17,68680016	-5,191610083
Moral Character	0b	0					
Affective Empathy	3,974817469	4,660513545	61,73439828	0,852871133	0,3970	-5,342213731	13,29184867
Immoral Character * Affective Empathy	12,18682098	0,760615562	189285,7755	16,02231349	0,0000	10,69603281	13,67760915
Moral Character * Affective Empathy	0b	0					

a. Dependent Variable: Character Morality Corrugator Response.
b. This parameter is set to zero because it is redundant.

Estimates of Fixed Effects Character: Affective Empathy*Immoral Character[a]

Parameter	Estimate	Std. Error	df	t	Sig.	95% Confidence Interval	
						Lower Bound	Upper Bound
Intercept	85,3466382	14,36846802	63,10629453	5,939856501	0,0000	56,63449248	114,0587839
Moral Character	11,43920512	3,183989055	1066,358317	3,592727525	0,0003	5,19161008	17,68680015
Immoral Character	0b	0					
Affective Empathy	16,16163843	4,660652077	61,74183853	3,467677519	0,0010	6,844352721	25,47892414
Moral Character * Affective Empathy	-12,18682098	0,760615562	189285,7755	-16,02231349	0,0000	-13,67760915	-10,69603281
Immoral Character * Affective Empathy	0b	0					

a. Dependent Variable: Character Morality Corrugator Response.
b. This parameter is set to zero because it is redundant.

Estimates of Fixed Effects Character: Transportability*Moral Character[a]

Parameter	Estimate	Std. Error	df	t	Sig.	95% Confidence Interval	
						Lower Bound	Upper Bound
Intercept	101,4400408	12,23718001	63,9261425	8,289494863	0,0000	76,99291947	125,8871622
Immoral Character	72,87553012	2,936067338	766,1527404	24,82079658	0,0000	67,11183868	78,63922156
Moral Character	0b	0					
Transportability	0,099847608	0,16129103	62,05755428	0,619052456	0,5381	-0,222562619	0,422257836
Immoral Character * Transportability	-0,651496392	0,026296924	186831,1443	-24,77462343	0,0000	-0,703037733	-0,59995505
Moral Character * Transportability	0b	0					

a. Dependent Variable: Character Morality Corrugator Response.
b. This parameter is set to zero because it is redundant.

Estimates of Fixed Effects Character: Transportability*Immoral Character[a]

Parameter	Estimate	Std. Error	df	t	Sig.	95% Confidence Interval	
						Lower Bound	Upper Bound
Intercept	174,315571	12,2369472	63,92112596	14,24502109	0,0000	149,8688777	198,7622642
Moral Character	-72,87553012	2,936067338	766,1527404	-24,82079658	0,0000	-78,63922156	-67,11183868
Immoral Character	0b	0					
Transportability	-0,551648783	0,16128593	62,04951982	-3,420315606	0,0011	-0,874049645	-0,229247921
Moral Character * Transportability	0,651496392	0,026296924	186831,1443	24,77462343	0,0000	0,59995505	0,703037733
Immoral Character * Transportability	0b	0					

a. Dependent Variable: Character Morality Corrugator Response.
b. This parameter is set to zero because it is redundant.

Supplementary Information B: Parameter Estimates of Individual Differences on Corrugator Response at Critical Event Segment

Highlighted are the *p* values for the effect of each individual differences measure on the average corrugator response for every condition

Estimates of Fixed Effects Character: Cognitive Empathy*Moral Positive[a]

Parameter	Estimate	Std. Error	df	t	Sig.	95% Confidence Interval	
						Lower Bound	Upper Bound
Intercept	92,46093735	15,50072325	63,51317153	5,964943431	0,0000	61,49011091	123,4317638
[moralposref=,00]	19,43406461	3,289906329	1510,236317	5,907178706	0,0000	12,98079485	25,88733437
[moralposref=1,00]	0b	0					
CE	4,211696337	4,30248923	61,86806176	0,97889759	0,3314	-4,389221946	12,81261462
[moralposref=,00] * CE	-2,1759794	0,716594616	190488,6938	-3,036555611	0,0024	-3,580487518	-0,771471283
[moralposref=1,00] * CE	0b	0					

a. Dependent Variable: Critical Event Corrugator Response.
b. This parameter is set to zero because it is redundant.

Estimates of Fixed Effects Character: Cognitive Empathy*Moral Negative[a]

Parameter	Estimate	Std. Error	df	t	Sig.	95% Confidence Interval	
						Lower Bound	Upper Bound
Intercept	112,6109196	15,50318332	63,55671428	7,263728827	0,0000	81,63558976	143,5862494
[moralnegref=,00]	-7,361595061	3,309463824	1438,360663	-2,224407171	0,0263	-13,85348774	-0,869702381
[moralnegref=1,00]	0b	0					
CE	3,048367469	4,302166043	61,85252878	0,708565741	0,4813	-5,551947802	11,64868274
[moralnegref=,00] * CE	-0,644494943	0,715123856	190550,5311	-0,901235412	0,3675	-2,046120406	0,757130519
[moralnegref=1,00] * CE	0b	0					

a. Dependent Variable: Critical Event Corrugator Response.
b. This parameter is set to zero because it is redundant.

Estimates of Fixed Effects Character: Cognitive Empathy*Immoral Negative[a]

Parameter	Estimate	Std. Error	df	t	Sig.	95% Confidence Interval	
						Lower Bound	Upper Bound
Intercept	124,787495	15,51307929	63,71097753	8,044018385	0,0000	93,79384862	155,7811414
[immoralnegref=,00]	-23,47140004	3,367705006	1317,35571	-6,969553447	0,0000	-30,07805055	-16,86474953
[immoralnegref=1,00]	0b	0					
CE	-2,473821326	4,30238106	61,85684502	-0,574988894	0,5674	-11,07455446	6,126911812
[immoralnegref=,00] * CE	6,682878362	0,715529816	190623,661	9,339762245	0,0000	5,280457232	8,085299492
[immoralnegref=1,00] * CE	0b	0					

a. Dependent Variable: Critical Event Corrugator Response.
b. This parameter is set to zero because it is redundant.

Estimates of Fixed Effects Character: Cognitive Empathy*Immoral Negative[a]

Parameter	Estimate	Std. Error	df	t	Sig.	95% Confidence Interval	
						Lower Bound	Upper Bound
Intercept	98,63158444	15,51106362	63,69976396	6,358789237	0,0000	67,64185961	129,6213093
[immoralposref=,00]	11,40721805	3,363460499	1324,041222	3,391512418	0,0007	4,808924913	18,00551119
[immoralposref=1,00]	0b	0					
CE	5,435483751	4,301980942	61,8551325	1,263483922	0,2112	-3,164454271	14,03542177
[immoralposref=,00] * CE	-3,8639102	0,715348529	190620,7902	-5,40143726	0,0000	-5,265976012	-2,461844388
[immoralposref=1,00] * CE	0b	0					

a. Dependent Variable: Critical Event Corrugator Response.
b. This parameter is set to zero because it is redundant.

Supplementary Information B: Parameter Estimates of Individual Differences on Corrugator Response at Critical Event Segment

Highlighted are the *p* values for the effect of each individual differences measure on the average corrugator response for every condition

Estimates of Fixed Effects Character: Affective Empathy*Moral Positive[a]

Parameter	Estimate	Std. Error	df	t	Sig.	95% Confidence Interval	
						Lower Bound	Upper Bound
Intercept	100,2819074	12,06771247	65,4534223	8,309935095	0,0000	76,184186	124,3796287
Other	-5,657660701	2,904619849	937,5842184	-1,947814515	0,0517	-11,35796959	0,042648182
Moral Positive	0b	0					
Affective Empathy	2,358952443	3,893940566	62,71893086	0,605800834	0,5468	-5,423149374	10,14105426
Other * Affective Empathy	5,779370387	0,671194752	189804,3227	8,610571472	0,0000	4,463844873	7,0948959
Moral Positive * Affective Empathy	0b	0					

a. Dependent Variable: Critical Event Corrugator Response.
b. This parameter is set to zero because it is redundant.

Estimates of Fixed Effects Character: Affective Empathy*Moral Negative[a]

Parameter	Estimate	Std. Error	df	t	Sig.	95% Confidence Interval	
						Lower Bound	Upper Bound
Intercept	95,81984239	12,07374213	65,56780736	7,936217399	0,0000	71,71087226	119,9288125
Other	0,349502899	2,932092349	893,5652533	0,119199144	0,9051	-5,405087112	6,104092909
Moral Negative	0b	0					
Affective Empathy	9,170310339	3,894113348	62,7147375	2,354916131	0,0217	1,387852984	16,95276769
Other * Affective Empathy	-3,322809387	0,669228242	189923,4199	-4,965136226	0,0000	-4,634480583	-2,011138192
Moral Negative * Affective Empathy	0b	0					

a. Dependent Variable: Critical Event Corrugator Response.
b. This parameter is set to zero because it is redundant.

Estimates of Fixed Effects Character: Affective Empathy*Immoral Negative[a]

Parameter	Estimate	Std. Error	df	t	Sig.	95% Confidence Interval	
						Lower Bound	Upper Bound
Intercept	96,37696748	12,08907757	65,8917637	7,972235012	0,0000	72,23960529	120,5143297
Other	-0,315682693	3,000988558	836,8027354	-0,105192901	0,9162	-6,206031865	5,57466648
Immoral Positive	0b	0					
Affective Empathy	6,538432644	3,895375244	62,78768969	1,678511628	0,0982	-1,246368796	14,32323408
Other * Affective Empathy	0,159942567	0,671648564	190099,7742	0,23813431	0,8118	-1,156472393	1,476357528
Immoral Negative* Affective Empathy	0b	0					

a. Dependent Variable: Critical Event Corrugator Response.
b. This parameter is set to zero because it is redundant.

Estimates of Fixed Effects Character: Affective Empathy*Immoral Positive

Parameter	Estimate	Std. Error	df	t	Sig.	95% Confidence Interval	
						Lower Bound	Upper Bound
Intercept	91,97440487	12,08827713	65,87414833	7,608561903	0,0000	67,83852018	116,1102896
Other	5,543351233	2,997072167	839,689928	1,84958884	0,0647	-0,339281539	11,42598401
Immoral Positive	0b	0					
Affective Empathy	8,606596984	3,895280129	62,78137497	2,20949372	0,0308	0,821970252	16,39122372
Other* Affective Empathy	-2,593948712	0,671248743	190142,4983	-3,86436286	0,0001	-3,909580031	-1,278317393
Immoral Positive * Affective Empathy	0b	0					

a. Dependent Variable: Critical Event Corrugator Response.
b. This parameter is set to zero because it is redundant.

Supplementary Information B: Parameter Estimates of Individual Differences on Corrugator Response at Critical Event Segment

Highlighted are the *p* values for the effect of each individual differences measure on the average corrugator response for every condition

Estimates of Fixed Effects Character: Sympathy*Moral Positive[a]

Parameter	Estimate	Std. Error	df	t	Sig.	95% Confidence Interval	
						Lower Bound	Upper Bound
Intercept	107,7450496	18,09353256	62,37443148	5,954892955	0,0000	71,58091177	143,9091874
Other	16,37965358	3,610842384	2199,749064	4,53624164	0,0000	9,298636412	23,46067075
Moral Positive	0b	0					
Sympathy	-0,101202263	4,62081127	61,17333186	-0,021901406	0,9826	-9,340556001	9,138151474
Other* Sympathy	-1,201359629	0,760771218	190886,033	-1,579133912	0,1143	-2,6924528	0,289733541
Moral Positive* Sympathy	0b	0					

a. Dependent Variable: Critical Event Corrugator Response.
b. This parameter is set to zero because it is redundant.

Estimates of Fixed Effects Character: Sympathy*Moral Negative[a]

Parameter	Estimate	Std. Error	df	t	Sig.	95% Confidence Interval	
						Lower Bound	Upper Bound
Intercept	134,4381408	18,08848295	62,33952669	7,432250736	0,0000	98,28369553	170,592586
Other	-19,23873284	3,615239204	2042,874207	-5,321565669	0,0000	-26,32867209	-12,14879359
Moral Negative	0b	0					
Sympathy	-2,860469039	4,618672424	61,09399512	-0,619327109	0,5380	-12,09578799	6,374849908
Other * Sympathy	2,484685337	0,754959444	190885,6529	3,291150746	0,0010	1,004983103	3,964387571
Moral Negative* Sympathy	0b	0					

a. Dependent Variable: Critical Event Corrugator Response.
b. This parameter is set to zero because it is redundant.

Estimates of Fixed Effects Character: Sympathy*Immoral Negative[a]

Parameter	Estimate	Std. Error	df	t	Sig.	95% Confidence Interval	
						Lower Bound	Upper Bound
Intercept	133,9144285	18,09571863	62,44772032	7,400337685	0,0000	97,74676052	170,0820965
Other	-18,49016792	3,678424896	1871,051215	-5,026653648	0,0000	-25,70441501	-11,27592083
Immoral Negative	0b	0					
Sympathy	-4,626668953	4,618489897	61,09227373	-1,001770937	0,3204	-13,86162818	4,608290273
Other * Sympathy	4,82738737	0,758370454	190889,5975	6,365473953	0,0000	3,34099964	6,313775101
Immoral Negative * Sympathy	0b	0					

a. Dependent Variable: Critical Event Corrugator Response.
b. This parameter is set to zero because it is redundant.

Estimates of Fixed Effects Character: Sympathy*Immoral Positive[a]

Parameter	Estimate	Std. Error	df	t	Sig.	95% Confidence Interval	
						Lower Bound	Upper Bound
Intercept	103,9927775	18,09414423	62,42961985	5,747316712	0,0000	67,82804921	140,1575059
Other	21,41069662	3,671187899	1871,974424	5,83208956	0,0000	14,21064526	28,61074798
Immoral Positive	0b	0					
Sympathy	3,588957228	4,618250021	61,08314883	0,777124931	0,4401	-5,645550208	12,82346466
Other * Sympathy	-6,1274006	0,757257743	190889,4597	-8,091565458	0,0000	-7,611607445	-4,643193755
Immoral Positive * Sympathy	0b	0					

a. Dependent Variable: Critical Event Corrugator Response.
b. This parameter is set to zero because it is redundant.

Supplementary Information B: Parameter Estimates of Individual Differences on Corrugator Response at Critical Event Segment

Highlighted are the *p* values for the effect of each individual differences measure on the average corrugator response for every condition

Estimates of Fixed Effects Character: Transportability*Moral Positive[a]

Parameter	Estimate	Std. Error	df	t	Sig.	95% Confidence Interval	
						Lower Bound	Upper Bound
Intercept	99,9262101	10,29351029	66,70358719	9,707690311	0,0000	79,37859916	120,473821
Other	28,95890057	2,693658317	688,8525118	10,75076983	0,0000	23,67013482	34,24766633
Moral Positive	0b	0					
Transportability	0,10169115	0,13470121	62,9305115	0,754938654	0,4531	-0,167493556	0,370875857
Other * Transportability	-0,23550067	0,023229729	187921,8835	-10,13789993	0,0000	-0,281030381	-0,189970959
Moral Positive * Transportability	0b	0					

a. Dependent Variable: Critical Event Corrugator Response.
b. This parameter is set to zero because it is redundant.

Estimates of Fixed Effects Character: Transportability*Moral Negative[a]

Parameter	Estimate	Std. Error	df	t	Sig.	95% Confidence Interval	
						Lower Bound	Upper Bound
Intercept	139,7060857	10,2987339	66,86806319	13,56536513	0,0000	119,1489812	160,2631902
Other	-24,08983862	2,721179815	671,2007537	-8,8527184	0,0000	-29,43288778	-18,74678946
Moral Negative	0b	0					
Transportability	-0,223070883	0,134701367	62,95858764	-1,656040227	0,1027	-0,492253552	0,046111786
Other* Transportability	0,197572541	0,023233044	188174,8872	8,503945432	0,0000	0,152036332	0,243108749
Moral Negative * Transportability	0b	0					

a. Dependent Variable: Critical Event Corrugator Response.
b. This parameter is set to zero because it is redundant.

Estimates of Fixed Effects Character: Transportability*Immoral Negative[a]

Parameter	Estimate	Std. Error	df	t	Sig.	95% Confidence Interval	
						Lower Bound	Upper Bound
Intercept	128,8626768	10,31737198	67,34751865	12,48987407	0,0000	108,2710693	149,4542843
Other	-9,708976286	2,800758482	627,9261098	-3,466552489	0,0006	-15,20896323	-4,208989338
Immoral Negative	0b	0					
Transportability	-0,175317356	0,134745072	63,03648122	-1,301104028	0,1980	-0,444580846	0,093946135
Other * Transportability	0,134983909	0,023250215	188723,2817	5,805705796	0,0000	0,089414047	0,180553771
Immoral Negative * Transportability	0b	0					

a. Dependent Variable: Critical Event Corrugator Response.
b. This parameter is set to zero because it is redundant.

Estimates of Fixed Effects Character: Transportability*Immoral Positive[a]

Parameter	Estimate	Std. Error	df	t	Sig.	95% Confidence Interval	
						Lower Bound	Upper Bound
Intercept	118,0153593	10,31423496	67,2759648	11,44198865	0,0000	97,42961241	138,6011063
Other	4,768089389	2,789703106	628,566525	1,709174492	0,0879	-0,710176796	10,24635557
Immoral Positive	0b	0					
Transportability	-0,002248864	0,134718456	62,99612184	-0,016693067	0,9867	-0,271462542	0,266964813
Other * Transportability	-0,095991147	0,023150774	188796,2997	-4,146347261	0,0000	-0,141366106	-0,050616188
Immoral Positive * Transportability	0b	0					

a. Dependent Variable: Critical Event Corrugator Response.
b. This parameter is set to zero because it is redundant.
